# The Prognostic Model of Pre-Treatment Complete Blood Count (CBC) for Recurrence in Early Cervical Cancer

**DOI:** 10.3390/jcm9092960

**Published:** 2020-09-13

**Authors:** Joseph J. Noh, Myong Cheol Lim, Moon-Hong Kim, Yun Hwan Kim, Eun Seop Song, Seok Ju Seong, Dong Hoon Suh, Jong-Min Lee, Chulmin Lee, Chel Hun Choi

**Affiliations:** 1 Division of Gynecologic Oncology, Department of Obstetrics and Gynecology, Samsung Medical Center, Sungkyunkwan University School of Medicine, 81 Irwon-Ro, Gangnam-gu, Seoul 06351, Korea; josephnoh.medicine@gmail.com; 2 Division of Tumor Immunology, Center for Gynecologic Center, and Center for Clinical Trials, Research Institute and Hospital and Cancer Control and Policy, Graduate School of Cancer Science and Policy, National Cancer Center, Goyang 10408, Korea; gynlim@gmail.com; 3 Department of Obstetrics and Gynecology, Korea Cancer Center Hospital, Korea Institute of Radiological and Medical Sciences, Seoul 01812, Korea; garymh@kcch.re.kr; 4 Department of Obstetrics and Gynecology, University of Ulsan College of Medicine, Asan Medical Center, Seoul 05505, Korea; medok74@gmail.com; 5 Medical Treatment Division, Gwangjin-gu Health Center, Seoul 05026, Korea; songsong2000@gmail.com; 6 Department of Obstetrics & Gynecology, CHA Gangnam Medical Center, CHA University, Seoul 06135, Korea; sjseongcheil@naver.com; 7 Department of Obstetrics and Gynecology, Seoul National University Bundang Hospital, Seongnam 13620, Korea; sdhwcj@snu.ac.kr; 8 Department of Obstetrics and Gynecology, Kyung Hee University Hospital at Gangdong, Kyung Hee University School of Medicine, Seoul 05278, Korea; kgo02@hanmail.net; 9 Department of Obstetrics and Gynecology, CHA Ilsan Medical Center, CHA University, Seoul 10414, Korea; morula3@gmail.com

**Keywords:** cervical cancer, prognosis, complete blood count, statistical model

## Abstract

The aim of the present study was to investigate the prognostic role of the pre-treatment complete blood count (CBC) profile as a predictive marker of survival, recurrence, and death in early stage squamous cell carcinoma and adenocarcinoma of the cervix. The pre-treatment CBC profiles of the patients from nine tertiary medical centers in South Korea who were treated surgically for early stage cervical cancer were reviewed. Statistical models by the Akaike’s information criterion (AIC) were developed using CBC profiles to calculate individuals’ risk scores for clinical outcomes. A total of 1443 patients were included in the study and the median follow-up was 63.7 months with a range of 3–183 months. Univariate analyses identified the components of CBC that were significantly related to clinical outcomes including white blood cell (WBC), hemoglobin, neutrophil, and platelet levels. The models developed using CBC profiles and the conventional clinical predictive factors provided individuals’ risk scores that were significantly better in predicting clinical outcomes than the models using the conventional clinical predictive factors alone. Pre-treatment CBC profiles including WBC, hemoglobin, neutrophil, lymphocyte, and platelet levels were found to be a potential biomarker for survival prognosis in early cervical cancer.

## 1. Introduction

Cervical cancer is the second most common malignancy in females worldwide and the leading cause of cancer-related deaths among women, especially in developing countries. In the year 2012, about 500,000 new cervical cancer cases were diagnosed, and 275,000 deaths were estimated to occur of this malignancy globally [1,2]. Patients with cervical cancer are prone to develop pelvic recurrence or distant metastasis. A 10–20% recurrence rate has been reported following primary surgery or radiotherapy in women with stage IB–IIA cervical cancer with no evidence of lymph node involvement; while up to 70% of patients with nodal metastases were reported to relapse [3,4,5,6]. It is therefore critical to define reliable prognostic factors that may help identify patients at high risk of recurrence. Furthermore, identifying patients who would not benefit from the current treatment modalities would allow physicians to offer them the opportunity to receive other types of treatment. The FIGO (International Federation of Gynecology and Obstetrics) staging system is one of the most commonly used predictors for survivals, and often determines patients’ treatment plans in the current clinical settings [7]. However, because it is based on clinical evaluation of the anatomic extent; the correlation between the FIGO staging and final histopathologic classification is not always accurate. The error rate is about 25% in patients with early stages (≤IIA) while it is 65% to 90% in patients with advanced stages (≥IIB) [8]. Hence, it is necessary to improve the FIGO staging system for more accurate prognosis in cervical cancer, thereby, allowing beneficial individualized treatment. 

A large number of translational research studies have revealed an association of various molecular biomarkers with clinical outcome in cervical cancer. Among them are peripheral blood cells. This association between clinical outcome and pre-treatment peripheral blood profile such as leukocytes, neutrophils, lymphocytes, and platelets has been observed not only in cervical cancer but also in other types of cancers as well. In addition, systemic-inflammation-based scores such as the Glasgow prognostic score (GPS), neutrophil lymphocyte ratio (NLR), and platelet lymphocyte ratio (PLR) have also been shown to have prognostic value [9].

In the present study, we retrospectively assessed the association between pre-treatment complete blood count (CBC) and clinical outcome in early cervical cancer patients. Our primary aim was to assess the potential prognostic value of CBC in addition to the FIGO staging system in early cervical cancer patients who underwent operative treatment. The secondary aim was to develop models utilizing these variables to predict patients’ individual clinical outcomes by calculating risk scores. In doing so, we evaluated overall survival, lymphatic recurrence, and hematogenous recurrence separately because each often requires different treatment modalities.

## 2. Materials and Methods

### 2.1. Patients

The medical records of the patients with early stage cervical cancer from January 2000 to December 2008 at nine Korean Gynecologic Oncology Group (KGOG) institutes were retrospectively reviewed. Approval from the institutional review board was obtained, and the requirement for informed consent was waived. Patients with pathologically confirmed cervical cancer, a clinical diagnosis of FIGO stage IB–IIA disease, who had undergone radical hysterectomy (type II or III) with pelvic and/or para-aortic lymphadenectomy, were examined. Patients who received neoadjuvant chemotherapy before surgery, those with a history of previous radiation therapy, those with cervical cancer found incidentally after simple hysterectomy, those with rare cell types, and those with metastatic cervical cancers were excluded. Patients who did not have a pre-treatment CBC profile within two weeks prior to their surgery were also excluded from analysis.

### 2.2. Clinical Management

Cervical cancer was staged according to the FIGO staging system using physical examination, chest radiography, intravenous pyelography, and abdominopelvic computed tomography (APCT) or pelvis magnetic resonance imaging (MRI) findings. Cystoscopy and colonoscopy were performed if bladder or rectal involvement was suspected. Tumor size was determined by clinical palpation or inspection, and in cases without data of clinical tumor size, it was measured using imaging modalities such as APCT or pelvic MRI. The operation records of laparotomy or laparoscopic radical hysterectomy with bilateral pelvic lymphadenectomy, with or without para-aortic lymphadenectomy, were reviewed to determine the appropriateness of the surgical procedure. Pathological reports included the description of histology, depth of stromal invasion (DSI), lymph-vascular space invasion (LVSI), the number of lymph nodes dissected, the number of nodes showing features of malignancy, parametrial involvement, and invasion of the resection margin.

Patients were followed-up according to the guidelines at each institution. The treatment options included no further treatment, radiation alone, concurrent platinum-based chemoradiation, and chemotherapy alone. In brief, adjuvant radiation therapy was started within 4–6 weeks after surgery using the conventional four-field technique. The radiation dose ranged from 40 Grays in 23 fractions to 50.4 Grays in 28 fractions (daily fractions of 1.8–2.0 Grays (5 fractions per week) over 4.5–6.0 weeks). The cisplatin-based concurrent chemotherapy regimens consisted of weekly cisplatin (40 mg/m^2^) for six cycles or FP (500 mg/m^2^ 5-fluorouracil (FU) + 50 mg/m^2^ cisplatin) every 3 weeks for three cycles. The systemic chemotherapy regimens consisted of CP (500 mg/m^2^ cyclophosphamide + 50 mg/m^2^ cisplatin), FP (500 mg/m^2^ 5-FU + 50 mg/m^2^ cisplatin), and TP (135 mg/m^2^ paclitaxel + 75 mg/m^2^ cisplatin).

At follow-up visits, patients received a physical examination, Pap smear, and serum squamous cell carcinoma (SCC) antigen level monitoring every 3 months for 2 years and then every 6 months for the next 3 years. Chest radiography and imaging modalities such as APCT or positron emission tomography/computed tomography (PET-CT) were performed every 6–12 months for the first 2 years and then annually for the next 3 years. Disease recurrence was defined as the histological presence of tumor cells by biopsy, appearance of new lesions on imaging modalities such as APCT or PET-CT that were suggestive of hematogenous recurrence, or any lymph node greater than 1 cm along the short-axis diameter on the APCT scan. When lymphatic recurrence was suspected, needle biopsy was often performed to confirm the malignant features of the lymph node. When needle biopsy was unavailable, PET-CT was performed to confirm high F-18 fluorodeoxyglucose (FDG) uptake. Hematogenous recurrence was defined as distant recurrence regardless of concurrent lymphatic recurrences.

### 2.3. Selection of Prognostic Variables for Survival and Recurrence

To identify variables predicting survival and recurrence, the following factors were evaluated: age, body mass index (BMI), FIGO stage, histology, parametrial invasion, LVSI, tumor size, DSI, lymph node metastasis (pelvic and para-aortic), resection margin invasion, pre-treatment CBC results within two weeks prior to the operation, and surgical methods. Disease-free survival (DFS) was defined as the time interval from surgery to the first evidence of recurrence or last follow-up. Overall survival (OS) was described as the time interval from diagnosis to date of death or last follow-up. DFS and OS were estimated using the Kaplan-Meier method. Survival curves were compared using the log-rank test.

### 2.4. Model Development

A Cox proportional hazards regression analysis was performed on each of the individual factors for DFS, OS, hematogenous recurrence, and lymphatic recurrence to estimate individuals’ risk scores. The Akaike’s information criterion (AIC) using stepwise and best-model options identified the best combinations as predictors. The AIC measures the relative quality of statistical models for a given set of data, thereby providing a means for model selection [10,11,12]. Conditional backward selection was used to select the final variables for constructing the models. This method is frequently used to identify predictive factors for prognosis in clinical research [13]. Hazard ratios (HRs) and 95% confidence intervals (CIs) were estimated.

### 2.5. Concordance Index (C-Index) Calculation

The predictive accuracy of the model was estimated by the concordance index (C-index). A C-index close to 1.0 reflects a model with near-perfect discrimination. In contrast, an index of 0.5 indicates that the model is capable of nothing better than random predictions [14]. The C-index of clinical outcome was calculated with the conventional clinical predictive factors including histologic tumor type, FIGO stage, DSI, lymph node metastasis, and parametrial invasion. The C-index was again calculated with the above variables and the pre-treatment CBC profiles added. These included hemoglobin, platelet, lymphocyte, neutrophil, NLR, and PLR. The two types of C-index were compared in each clinical outcome. Cross validation based on bootstrap resampling resulted in ranges of C-index. This analysis was performed with the patients who did not have any missing clinical data or pre-treatment CBC profile.

### 2.6. Statistical Analysis

Statistical analysis was performed using R 3.5.0. (R Foundation for Statistical Computing, Vienna, Austria; http://www.R-project.org/). A *p*-value of less than 0.05 was considered significant.

## 3. Results

### 3.1. Patient Characteristics and Univariate Analysis of Pre-Treatment CBC

From a database of 1553 patients, 1443 patients satisfied the eligibility criteria. Descriptive statistics of the patients are described in Table 1. During the median follow-up period of 63.7 months (range: 3.0–183.3 months), 134 patients experienced recurrences and 72 cancer-related deaths occurred. Among the 1443 patients, 675 patients received adjuvant treatment after surgery. Univariate analysis indicated that hemoglobin and platelet levels were related to overall recurrence (DFS) (Supplementary Table S1). When analyzed separately by the types of recurrence, platelet levels were shown to have an association with hematogenous recurrence (Supplementary Table S2).

### 3.2. Model Development

The models to calculate risk scores for clinical outcomes were developed by the AIC using stepwise and best-model options with pre-treatment CBC. The multivariate regression analysis revealed that lymphocyte and platelet levels were significantly related to overall recurrence (DFS) (Table 2) while white blood cell (WBC), neutrophil, and platelet levels were associated with hematogenous recurrence (Table 3). The AIC models were established based upon the results of multivariate analysis. For example, the model to predict overall recurrence (DFS) included lymphocyte and platelet levels as following: RS (risk score) = (0.029 × Platelet) – (0.299 × Lymphocyte). The same calculations were conducted to build models to predict hematogenous recurrence: RS = (0.507 × Neutrophil) + (0.048 × Platelet) – (0.505 × WBC). We were not able to build a model to predict lymphatic recurrence because no variables showed statistically significant association with it.

**Figure 1:**
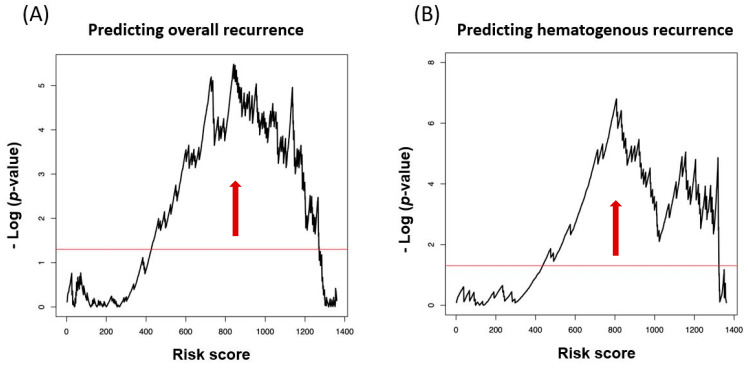
(**A**) Distribution of risk scores calculated by the built models to predict disease-free survival (DFS) and (**B**) hematogenous recurrence. The red horizontal lines represent the negative log of 0.05. The risk scores distributed above the red lines demonstrate statistical significance. The red vertical arrows indicate the risk scores with the smallest *p*-values in each model. These risk scores were used to dichotomize high-risk-score group vs. low-risk-score group in further analysis described in Section 3.3.

The calculated risk scores predicted recurrence (overall and hematogenous) with good performance. Negative log (*p*-value) of log rank test showed significance (area over the red line) for most cutoff points (Figure 1).

### 3.3. Risk Score as a Prognostic Marker in Each Traditional Risk Subgroup

Further subgroup analyses of risk scores for predicting each clinical outcome were conducted. The patients were categorized into one of three groups—no risk, intermediate risk, and high risk for recurrence. Intermediate risk factors for recurrent disease included (1) large tumor size (greater than 2 cm in diameter), (2) cervical stromal invasion to the middle or deep one-third, and (3) lymph-vascular space invasion. High risk factors for recurrent disease included (1) positive or close margins (less than 5 mm), (2) positive lymph nodes, and (3) microscopic parametrial involvement.

**Figure 2:**
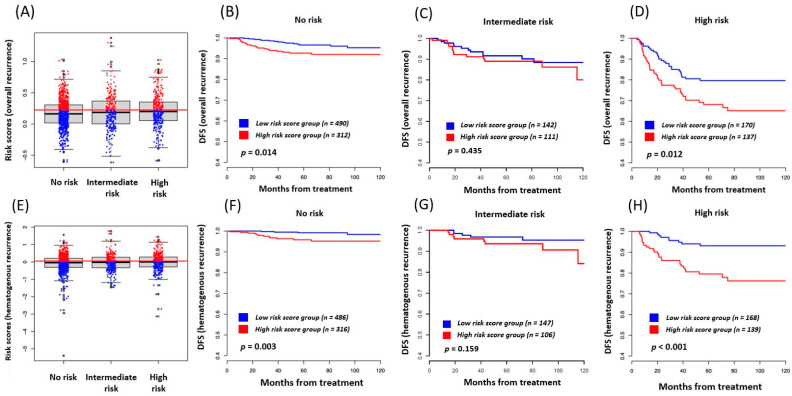
(**A**) Box-plots of risk scores calculated to predict DFS by the built model. Patients were classified according to the conventional risk factors as described in the text (no risk factors, intermediate risk factors, and high risk factors), (**B**) patients were divided into two groups in each classification according to their calculated risk scores. The Kaplan–Meier analysis of DFS of the patients with no risk factors is represented, (**C**) Kaplan–Meier analysis of DFS of the patients with intermediate risk factors is represented, (D) Kaplan–Meier analysis of DFS of the patients with high risk factors is represented, (**E**–**H**) the same analyses were performed to predict hematogenous recurrence, which also demonstrated similar patterns as the analyses for DFS.

The risk scores of the patients according to the above classifications are box-plotted in Figure 2A. The DFS of the patients without traditional risk factors was divided into two according to their risk scores calculated by the built model. It was performed by dichotomizing the patients at the lowest *p*-value (indicated by the red vertical arrows in Figure 1), and they were named as the low-risk-score group and the high-risk-score group accordingly. The two groups were prognostically different in Kaplan–Meier curves (Figure 2B). The same analysis was conducted for the patients with intermediate- and high-risk groups in Figure 2C,D. The risk groups created by the CBC-based models further predicted DFS with a large difference in survival in the patients with no risk factors and high risk factors. The analysis did not show significant differences in the patients with intermediate risk factors. The same analyses were performed for predicting hematogenous recurrence, and they are described in Figure 2E–H, demonstrating similar patterns.

### 3.4. Concordance Index (C-Index) Calculation

**Figure 3:**
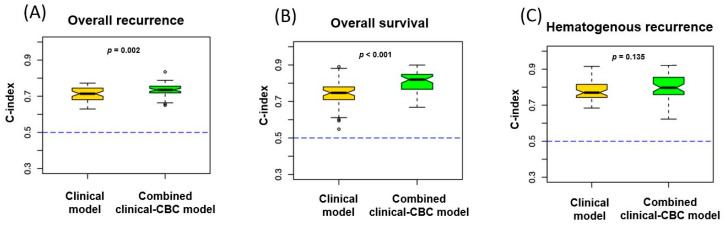
(**A**) The concordance index (C-index) of overall recurrence, (**B**) overall survival, and (**C**) hematogenous recurrence calculated each by the known prognostic factors only and the known prognostic factors with CBC profiles added. The yellow boxes-and-whiskers represent the C-index calculated by the known prognostic factors only and the green boxes-and-whiskers represent the C-index calculated by the known prognostic factors with CBC profiles added. The blue dotted lines represent the C-index value of 0.5, scores under which indicate that the model is no better than chance results. Cross validation based on bootstrap resamplinggenerated ranges of C-index represented in each figure.

When pre-treatment CBC profiles were added to the conventional clinical variables to predict clinical outcomes, the C-index values increased significantly for predicting overall recurrence and overall survival (Figure 3A,B). However, the addition of pre-treatment CBC profiles to the conventional clinical variables for predicting hematogenous recurrence did not contribute any statistically significant increase in predictive ability.

## 4. Discussion

The present study showed that WBC, hemoglobin, lymphocyte, and platelet levels measured pre-operatively have predictive value for prognosis in early cervical cancer. These variables allowed us to build models based on stepwise and backward reducing methods (Akaike’s information criterion, AIC) and to calculate each individual’s risk scores for predicting overall recurrence, hematogenous recurrence, lymphatic recurrence, and overall survival. The discriminatory capacities of the models were statistically sufficient to identify subsets of patients who were at high risk for poor clinical outcomes, and these predictions were in accordance with other already known prognostic factors in early cervical cancer, i.e., the FIGO stages.

Pre-treatment CBC has become one of the predictors of prognosis in many types of solid cancers. These include colorectal cancer, hepatocellular carcinoma, pancreatic carcinoma, and breast cancer [15,16,17,18]. This prognostic value has also been observed in gynecologic malignancies including ovarian cancer, endometrial cancer, and cervical cancer [19,20,21]. Although variations exist in the results of previous studies, they can be summarized as following: (1) high levels of pre-operative WBC predict poor prognosis, (2) high levels of pre-operative monocyte count predict poor prognosis, (3) low levels of pre-operative lymphocyte count predict poor prognosis, (4) high levels of pre-operative platelet count predict poor prognosis, and (5) low levels of pre-operative hemoglobin predict poor prognosis.

The exact biologic mechanisms by which peripheral blood cells interact with tumor progression and metastasis are still under investigation. However, many clinical reviews and experimental reports are accumulating to implicate that it comprises a cascade of steps that involve the interaction between the tumor and the host-derived stromal microenvironment, which includes factors that support angiogenesis and inflammation [22,23]. Increased WBC is caused by the upregulated production of hematopoietic growth factors, including granulocyte colony-stimulating factor (G-CSF), interleukin (IL)-1, IL-6, and tumor necrosis factor (TNF)-alpha [24,25,26,27,28]. Among these cytokines, G-CSF plays a crucial role in granulopoiesis by stimulating the proliferation, survival, and neutrophilic differentiation of hematopoietic progenitor cells [29]. It was reported that G-CSF is produced by tumor cells themselves, and an increasing number of studies have reported that G-CSF-producing malignancies including cervical cancer were associated with a poor clinical outcome [30,31,32,33]. Increased neutrophils have been considered to be the primary source of circulating vascular endothelial growth factor, which play a critical role in tumor-associated angiogenesis. It produces inflammatory cytokines such as TNF-alpha and IL-1 and provides a favorable microenvironment for tumor [34]. Decreased lymphocytes exert an adverse effect in cancer-specific immune response [35]. It has been shown that an increased infiltration of lymphocytes into tumor tissue is associated with good prognosis [36]. Conversely, decreased lymphocytes represent low immune competence of the host in tumor environment. Low hemoglobin represents hypoxic tissue conditions. Hypoxia confers resistance to ionizing radiation as well as to many anticancer drugs and triggers proteome and genomic changes that induce tumor progression [37,38,39,40]. The increase in platelet counts is due to tumor-secreted cytokines that play a role in stimulating megakaryocyte growth and thrombopoiesis. IL-6, in particular, acting as an autocrine growth factor, is overproduced in a variety of malignancies [41]. Platelets also enhance tumor metastasis by expressing immunoregulatory proteins including the glucocorticoid-induced TNF-related protein to protect tumor cells from the host’s immune system [42,43,44].

In our study, we were able to develop statistical models to calculate each patient’s risk scores for recurrence. The risk of recurrence in a patient exists as a continuous spectrum influenced by multiple, interrelated elements. This may explain why individual pathologic features or varying combinations of such features had diverse predictive values among different investigators. In contrast to traditional prognostic systems, which allocate patients into discrete risk groups, a statistical predictive model such as the one developed in the present study can generate a numerical probability of a clinical outcome. In recent years, statistical prediction models have been developed across many other types of cancer [45,46,47,48,49]. These are becoming widely used for cancer prognosis because of their ability to provide a single numerical estimate of the probability of an event, such as death or recurrence, that is tailored to the profile of an individual patient [50]. It is not surprising that the predictive model performs better than the staging system because it is better able to account for heterogeneity in tumor and patient characteristics. Considering the fact that the C-index of the Gail model, one of the most widely used risk assessment tools for predicting breast cancer, is 0.67, the increase of C-index shown in the present study by the addition of CBC profiles to the conventional clinical factors in cervical cancer is noteworthy. Another potential benefit of the predictive statistical model is that it is readily modifiable by adding other prognostic factors. Therefore, the models developed in the present study may aid clinicians in determining more aggressive treatment methods for those who acquire high risk scores for poor clinical outcomes.

The present study carries the following limitations. First it is a retrospective study. This structure of the study does not exclude the possibility that the prognosis was affected by different therapeutic strategies such as the types of hysterectomy or types of adjuvant therapies. A well-designed, prospective study with a larger number of patients with cervical cancer who underwent radical surgery is needed. Second, C-reactive protein (CRP), an inflammatory marker, was not evaluated in the present study. Many authors have observed an association between elevated serum CRP levels and poor prognosis in cancers such as colorectal [51,52], lung [53], and head and neck cancers [54,55]. It would be more useful if models for predicting clinical outcomes in cervical cancer were developed not only with CBC profiles but also with other potential biomarkers such as CRP. Third, differences may exist in host’s immune condition between human papillomavirus (HPV)-negative cervical cancer patients and HPV-positive cervical cancer patients. Some heterogeneity may be found in this study because we included cervical cancer patients both with and without HPV infection.

In conclusion, we have shown that pre-treatment CBC can be utilized in addition to the existing clinical predictive factors to develop better statistical models in early cervical cancer. We believe that our results suggest that it is possible to identify high-risk patients who would not benefit from conventional treatment by performing a simple blood test, providing the physician the opportunity to offer patients other types of individualized treatment options.

## Figures and Tables

**Table 1:** Descriptive statistics of the patients.

	Total (N = 1443)	
Age (years)	48	(41–57)
FIGO stage		
IB1/IIA	1274	(88.3%)
IB2	167	(11.6%)
Missing data	2	(0.1%)
Histology		
Squamous cell carcinoma	1089	(75.5%)
Adenocarcinoma	277	(19.2%)
Adenosquamous carcinoma	75	(5.2%)
Missing data	2	(0.1%)
Lymphovascular space invasion		
Negative	791	(54.8%)
Positive	562	(38.9%)
Missing data	90	(6.2%)
Depth of stromal invasion		
Superficial 1/3	445	(30.8%)
Middle 1/3	380	(26.3%)
Deeper 1/3	577	(40.0%)
Missing data	41	(2.8%)
Depth of stromal invasion		
Superficial 1/2	642	(44.5%)
Deeper 1/2	758	(52.5%)
Missing data	43	(3.0%)
Lymph node metastasis		
No lymph node metastasis	1177	(81.6%)
Pelvic lymph node metastasis	239	(16.6%)
Para-aortic lymph node metastasis	22	(1.5%)
Missing data	5	(0.3%)
Parametrial invasion		
Negative	1323	(91.7%)
Positive	116	(8.0%)
Missing data	4	(0.3%)
Resection margin free		
Resection margin negative	1381	(95.7%)
Resection margin (carcinoma in situ)	16	(1.1%)
Resection margin (cancer)	44	(3.0%)
Missing data	2	(0.1%)
Tumor size (cm)	2.7	(1.6–4.0)
White blood cell (×1000 cells/mm^3^)	6.2	(5.2–7.5)
Lymphocyte (×10%)	1.9	(1.5–2.3)
Monocyte (×10%)	0.4	(0.3–0.5)
Neutrophil (×1000 cells/mm^2^)	3.6	(2.7–4.6)
Glucose (milligrams/deciliter)	100.0	(91.0–111.0)
Hemoglobin (grams/deciliter)	12.7	(11.8–13.4)
Platelet (×10,000 cells/mm^2^)	25.2	(21.5–29.8)
Neutrophil-lymphocyte ratio (NLR)	1.9	(1.4–2.6)
Platelet-lymphocyte ratio (PLR)	13.5	(10.6–17.0)

FIGO: International Federation of Gynecology and Obstetrics.

**Table 2:** Results of multivariate analysis to predict overall recurrence (disease free survival, DFS).

	Hazard Ratio	95% Confidence Interval		*p*-Value
		Lower Limit	Upper Limit	
Lymphocyte	0.74	0.55	0.99	0.046
Platelet	1.03	1.01	1.05	0.009

**Table 3:** Results of multivariate analysis to predict hematogenous recurrence.

	Hazard Ratio	95% Confidence Interval		*p*-Value
		Lower Limit	Upper Limit	
WBC ^§^	0.60	0.41	0.88	0.010
Neutrophil	1.66	1.11	2.49	0.014
Platelet	1.05	1.02	1.08	0.002

^§^ WBC: white blood cell.

## Supplementary Materials

The following are available online at https://www.mdpi.com/2077-0383/9/9/2960/s1, Table S1. Univariate analysis of disease-free survival by the Cox proportional hazard model. Table S2. Univariate analysis of hematogenous recurrence by the Cox proportional hazard model.
