# Supplementary material for: The Prognostic Model of Pre-Treatment Complete Blood Count (CBC) for Recurrence in Early Cervical Cancer
Source: J Clin Med. 2020 Sep 13;9(9):2960. doi: 10.3390/jcm9092960 (PMC7563514; doi:10.3390/jcm9092960)
Supplement: Supplementary file 1 [file jcm-09-02960-s001.pdf]

Supplementary Table S1. Univariate analysis of disease-free survival by the Cox proportional hazard model.

	Hazard ratio	95% confidence interval		*p*-value
		Lower limit	Upper limit	
**Age**	**0.93**	0.79	1.08	0.335
FIGO stage	2.43	1.62	3.65	< 0.001
Histology	2.21	1.56	3.11	< 0.001
Lymphovascular space invasion	3.52	2.43	5.10	< 0.001
Depth of stromal invasion (Divided by 3)	2.76	1.48	5.16	< 0.001
Depth of stromal invasion (Divided by 2)	3.00	2.00	4.51	< 0.001
Lymph node metastasis	3.58	2.50	5.13	< 0.001
Parametrial invasion	3.64	2.41	5.49	< 0.001
Resection margin free	0.87	0.12	6.24	0.891
Tumor size	1.33	1.22	1.44	< 0.001
White blood cell	0.97	0.89	1.05	0.471
Lymphocyte	0.80	0.60	1.07	0.132
Monocyte	0.63	0.13	3.01	0.566
Neutrophil	1.01	0.92	1.10	0.901
Glucose	1.00	0.99	1.00	0.307
Hemoglobin	0.85	0.77	0.93	< 0.001
Platelet	1.03	1.01	1.05	0.016
Neutrophil-lymphocyte ratio (NLR)	1.04	0.97	1.11	0.289
Platelet-lymphocyte ratio (PLR)	1.00	1.00	1.00	0.729

Supplementary Table S2. Univariate analysis of hematogenous recurrence by the Cox proportional hazard model.

	Hazard ratio	95% confidence interval		*p*-value
		Lower limit	Upper limit	
**Age**	**0.97**	0.78	1.22	0.821
FIGO stage	1.86	1.00	3.49	0.051
Histology	4.04	2.47	6.60	< 0.001
Lymphovascular space invasion	3.47	2.03	5.93	< 0.001
Depth of stromal invasion (Divided by 3)	6.60	1.93	22.51	0.003
Depth of stromal invasion (Divided by 2)	3.79	2.02	7.08	< 0.001
Lymph node metastasis	4.06	2.43	6.76	< 0.001
Parametrial invasion	5.34	3.13	9.12	< 0.001
Resection margin free	1.94	0.27	14.03	0.511
Tumor size	1.40	1.25	1.58	< 0.001
White blood cell	0.97	0.86	1.09	0.595
Lymphocyte	0.77	0.51	1.16	0.214
Monocyte	0.31	0.03	3.21	0.326
Neutrophil	1.04	0.92	1.17	0.578
Glucose	1.00	0.98	1.01	0.469
Hemoglobin	0.88	0.76	1.02	0.081
Platelet	1.04	1.01	1.07	0.005
Neutrophil-lymphocyte ratio (NLR)	1.05	0.95	1.15	0.355
Platelet-lymphocyte ratio (PLR)	1.00	1.00	1.00	0.869
